# A Cross-Sectional Study to Assess Knowledge, Attitude, and Practices Towards COVID-19 Pandemic Amongst Pregnant Women and Healthcare Staff at a Periurban Teaching Hospital in Haryana

**DOI:** 10.7759/cureus.36458

**Published:** 2023-03-21

**Authors:** Auditi Narayan, Atindra Narayan, Bhumika Singhal, Poonam Taneja

**Affiliations:** 1 Obstetrics and Gynaecology, SGT Medical College, Hospital and Research Institute, Haryana, IND; 2 Medicine, Dr. Baba Saheb Ambedkar Medical College and Hospital, Delhi, IND

**Keywords:** questionnaire, practice, attitude, knowledge, healthcare workers, pregnancy, covid-19 vaccination

## Abstract

Objective: The objective is to assess knowledge, attitude, and practices towards the COVID-19 pandemic amongst pregnant women and healthcare staff at a periurban teaching hospital in Haryana, India.

Methods: This was a single centre questionnaire-based cross-sectional analysis regarding COVID-19 which was conducted at a periurban teaching hospital in Haryana, India, amongst 300 participants which included pregnant women and healthcare staff involved in managing them. They were assessed for demographic details and KAP scores (knowledge-14 questions, attitude-9 questions, and practice-14 questions). Analysis of data was done using IBM Statistical Package for the Social Sciences (SPSS) version 25.0.

Results: Participants in the present study had an overall adequate mean score of knowledge (22.54 ± 5.22) and were following correct practices (mean score 23.91 ± 6.72) to prevent COVID-19. The overall correlation of knowledge and practice also shows a positive correlation (0.939, p=<0.0001).

Conclusion: This study demonstrated that the majority of the pregnant women and healthcare workers involved in the management of pregnant women had adequate knowledge and a positive attitude towards tackling COVID-19. They were following correct practices and taking necessary steps for the prevention of the disease. They had adequate knowledge regarding vaccination for pregnant females.

## Introduction

The coronavirus disease 2019 (COVID-19) pandemic has had a significant impact on all aspects of life, including a risk of infection during pregnancy complicating routine pregnancy care and delivery [1]. It is agreed that an effective vaccine may be able to slow or even halt the rapid spread of the COVID-19 virus. It is the most anticipated resolution in the antenatal population as well. The massive spread of misinformation brought on by the COVID-19 pandemic, as well as the easy access to various media channels and social media along with a history of chronic disease, source of vaccine knowledge, and education level, are the factors that affect the willingness to accept the vaccine amongst pregnant females and even the healthcare staff responsible for their care.

The COVID-19 pandemic is an ongoing threat and measures need to be taken to keep it in check. A complete course of vaccination for all those who are eligible for the same is currently thought to be the most effective preventive measure [2]. Despite the evidence proving the safety and efficacy of vaccines against COVID-19 as well as recommendations from various professional organizations, there is still hesitancy regarding COVID-19 vaccination during pregnancy [3-6].

The current level of awareness amongst pregnant women regarding COVID-19 pandemic is inadequately studied, so this analysis was aimed to evaluate the KAP of pregnant women and healthcare staff involved in managing them at a periurban teaching hospital in Haryana, India regarding their awareness and practices in fighting COVID-19 pandemic. This will help in designing appropriate behaviour change communication campaigns for improving the standards of care available to the general population.

## Materials and methods

Study design

This was a cross-sectional validated questionnaire-based study conducted at the Obstetrics and Gynaecology department of a periurban tertiary care teaching hospital in Haryana, North India. Approval for this study was obtained from the Institutional Ethical Committee (Reference number: SEC/FMHS/F/17/12/21-7).

The sample size included 100 consecutive consenting pregnant women, 100 doctors and medical interns working in the same hospital and 100 healthcare workers, other than doctors (nurses, technicians in radiology and hospital labs, and patient care attendants working in the hospital wards) in the same institute, totalling 300. All pregnant women attending the antenatal OPD were invited to participate in the study.

All consecutive pregnant women willing to participate in the study were recruited after taking informed consent till 100 pregnant women were recruited. They were assessed using a pre-approved questionnaire on their demographic profile and KAP scores (knowledge - 14, attitude - 9, practice - 14 questions) regarding their views on the COVID-19 pandemic and vaccine acceptability. The questionnaire was self-prepared using the recommendations from World Health Organisation on COVID-19 in pregnancy [7]. It was reviewed and validated by all the authors and scientific committee members. In the case of illiterate pregnant women, the information was filled in by a member of the team.

The questionnaire comprises two sections - demographic details and KAP. Demographic details included the name, age, residence, educational qualifications, and profession. KAP included 14 questions for the evaluation of knowledge, 9 questions on attitude, and 14 questions related to practice.

The knowledge was evaluated through a set of 14 questions on epidemiology, transmission, clinical features, prevention, treatments available, the effect of COVID-19 on mother and foetus, mode of delivery in COVID-19 mothers, and feeding options for the baby. To assess the degree of knowledge of each individual, a scoring system was applied. Depending on the question and response, a score of 2 was given for the correct response, 1 was given to a response that was correct to some extent and 0 was given for the wrong response. If their score was equal to or more than the median score the knowledge was considered adequate.

The Attitude was measured by a set of 9 questions which evaluated their behaviour towards COVID-19 disease, seriousness in following preventive measures, concerns if they contracted the disease, place of delivery and quarantine, and their confidence in victory in the fight against COVID-19.

Practices amongst participants were scored using 14 questions based on adherence to preventive measures, frequency of hand washing, eating habits, behavioural changes towards family members, consumption of herbal medicines, coughing etiquettes, and preferred feeding methods for the baby.

The score range of knowledge and practice were 2-18 and 2-19 respectively. The duration of the study is three months (July to September 2021).

Inclusion criteria

1. Consenting pregnant women attending antenatal OPD at SGT hospital.

2. Consenting pregnant women admitted to the Obstetrics ward of SGT hospital.

3. Doctors attending to antenatal women in Obstetrics OPD.

4. Nurses/ other healthcare professionals attending to pregnant women in Obstetric OPD/labour room/obstetric ward/laboratories in SGT hospital.

Exclusion criteria

1. Not willing to participate in the study.

2. Haemodynamically unstable patients.

Analysis of data was done using IBM Statistical Package for the Social Sciences (SPSS) version 25.0 using appropriate statistical tests.

## Results

Table 1 shows the demographic distribution of the study participants that included their age, religion, level of education, and occupation. Most of the responders 126 (42%) were in the age group of 18-25 years while 105 (35%) were in the age group of 26-30 years. 264 (88%) participants were Hindus while 27 (9%) were Muslims. The majority of respondents have completed secondary education 114 (38%) while 96 (32%) responders had obtained a primary education. Tables 2-4 show the responses to the questionnaire divided into three sections, i.e., knowledge (14), attitude (9), and practice (14). The results show that 93% of the people are aware that COVID-19 is a communicable disease, 62% feel they are at risk of it and 49% feel that they can contract COVID-19. 83% think that COVID-19 infection can also pose an additional risk to the pregnancy. Most of them (41%) think that COVID-19 can lead to a complication during the antenatal period, delivery, or foetal complications. 168 (56%) of the subjects feel that vaccination is the required cure for COVID-19. 252 (84%) respondents feel that either Covishield or Covaxin is safe in pregnancy however only 48% of the responders have been vaccinated and amongst the pregnant population in the study numbers are very low (34%).

**Table 1:** Characteristics of the study population

Parameter	n=300 (%)
Range (Years)	
<18	6 (2%)
18-25	126 (42%)
26-30	105 (35%)
31-40	75 (15%)
41-50	15 (5%)
51-60	3 (1%)
>60	3 (1%)
Religion n (%)	
Hindu	264 (88%)
Muslim	27 (9%)
Sikh	3 (1%)
Christian	6 (2%)
Education level n (%)	
Primary education and below	96 (32%)
Secondary education	114 (38%)
Graduation and higher	90 (30%)
Occupation n (%)	
Health care related	186 (62%)
Non-health care related	114 (38%)

**Table 2:** Responses of the Knowledge questionnaire

Sr. no.	Questions	Responses	Score	N	(%)
K1.	What type of disease is COVID-19 ?	1. Non-communicable	0	3	1%
		2. Communicable	2	279	93%
		3. Both	1	15	5%
		4. Not answered	0	3	1%
K2.	Does COVID-19 pose additional risk of infection to the pregnancy?	1. Yes	0	249	83%
		2. Don’t know	1	33	11%
		3. No	2	18	6%
K3.	If yes, then how?	1. The foetus will be affected	NA	15	5%
		2. Delivery will be complicated		12	4%
		3. Antenatal complications are associated		18	6%
		4. All of the above		123	41%
		5. More research is required		132	44%
K4.	Are you at risk for COVID-19?	1. Yes	NA	186	62%
		2. No		114	38%
K5.	What is the treatment of cure available for COVID 19?	1. Vaccine	1	168	56%
		2. Homoeopathic	1	3	1%
		3. Allopathic	1	45	15%
		4. No proven treatment yet	2	75	25%
		5. Not answered	0	9	3%
K6.	Is Covishield/ Covaxin safe in pregnancy?	1. Yes	NA	252	84%
		2. No		48	16%
K7.	Have you been vaccinated with COVID-19 vaccine?	1. Yes	NA	144	48
		2. No		156	52%
K8.	If no, why not?	1. Fear of going to hospital		88	29.30%
		2. Fear of side-effects		210	70%
		3. Family against vaccination during pregnancy		2	0.70%
K9.	What are the treatment available for COVID-19?	1. Hydroxychloroquine	1	51	17%
		2. Vaccine	1	240	80%
		3. Lemon	1	6	2%
		4. Ginger/garlic	1	3	1%
		5. Not answered	0	0	0
K10.	What should be the mode of delivery in COVID-19 patient?	1. Normal	NA	234	78%
		2. LSCS		57	19%
		3. Don’t know		9	3%
K11.	Which of the following are symptoms suggestive of COVID-19?	1. Fever	1	195	65%
		2. Cough	1	180	60%
		3. Breathlessness	1	168	56%
		4. Myalgia/weakness	1	135	45%
		5. Gi symptoms	1	96	32%
		6. Loss of taste/smell	1	222	74%
K12.	What are the feeding options for the baby born to COVID-19 mother?	1. Breastfeeding	1	159	53%
		2. Expressed breast milk	1	66	22%
		3. Formula-feed	1	74	24%
K13.	What precautions should you ensure if you are tested COVID-19 positive, and you are with the baby?	1. Hand hygiene	1	276	92%
		2. Wear mask	1	9	3%
		3. Disinfecting/cleaning surfaces with which you have been contacting with	1	6	2%
		4. All of the above	2	6	2%
		5. None of the above	0	3	1%
K14.	If a pregnant female has been close contact with someone infected with the COVID-19 virus, what should be the period of isolation for observation of symptoms?	1. 8-14days	2	195	65%
		2. 7 days or more	1	99	33%
		3. Less than 7 days	0	6	2%

**Table 3:** Responses of the Attitude questionnaire

Serial no.	Question	Responses	N	(%)
A1.	Do you think you can have COVID-19?	1. Yes	147	49%
		2. No	63	21%
		3. Do not know	90	30%
A2	Do you think your baby can be affected?	1. Yes	123	41%
		2. No	84	28%
		3. Do not know	93	31%
A3	If you develop symptoms suggestive of COVID-19, whom would you	1. Health personnel	156	52%
	communicate about it first?	2. Parents	93	31%
		3. Husband	21	7%
		4. Nobody	30	10%
A4	What will you do if you suspect that you have COVID-19?	1. Visit health care facility	219	73%
		2. Visit traditional/ local healer	6	2%
		3. Do not believe in treatment	18	6%
		4. Local chemist	9	3%
		5. Self assessment	48	16%
A5	What concerns you the most if you are diagnosed with COVID-19?	1. Fear of transmitting it to baby/family members	93	31%
		2. Social stigma	105	35%
		3. Cost of treatment	39	13%
		4. Fear of death	27	9%
		5. I am confident that it will get cured	36	12%
A6	On a scale of 1–5, how seriously one should follow preventive steps?	1	9	3%
		2	6	2%
		3	27	9%
		4	39	13%
		5	219	73%
A7	Where should the pregnant females with COVID-19 get delivered?	1. Home	18	6%
		2. Hospital	264	88%
		3. Quarantine centre	12	4%
		4. Does not matter	6	2%
A8	Suppose you test COVID-19 positive and are asymptomatic, where would you opt to get quarantined?	1. Home	237	79%
		2. Hospital	27	9%
		3. Quarantine centre	24	8%
		4. Anywhere	12	4%
A9	Do you think that India the battle against COVID-19 is over?	1. Yes	27	9%
		2. No	258	86%
		5. Do not know	15	5%

**Table 4:** Responses of the Practice questionnaire

Serial no.	Question	Response	Score		n(%)
P1	What precautions are you practicing in order to prevent contracting and spreading of COVID-19?	1. Wearing mask	1	255	85%
		2. Avoid crowded places	1	249	83%
		3. Avoid handshaking/social disrance	1	240	80%
		4. Washing vegetables before storing them	1	156	52%
		5. All of the above	1	267	89%
P2	Has there been any change in the frequency of handwashing to prevent contracting and spreading of COVID-19?	1. Increased	2	264	88%
		2. Decreased	0	6	2%
		3. Same as before	1	30	10%
P3	Did you visit a mall/club/recreational activity during festivals like diwali christmas?	1. Yes	0	249	83%
		2. No	1	51	17%
P4	How many times in a month do you visit local market?	1. Almost never do online shopping	NA	75	25%
		2. When essential		72	24%
		3. Once a week for recreational purpose		51	17%
		4. Twice a week essential shopping		42	14%
		5. As often we can daily		60	20%
P5	Suppose you are in the first trimester during the ongoing COVID-19 pandemic; would you like to continue pregnancy?	1. Yes, I want to Continue pregnancy	NA	189	63%
		2. Don’t know		87	29%
		3. I do not want to continue pregnancy		24	8%
P6	Would you visit hospital for regular antenatal checkup	1. Yes	1	252	84%
		2. No	0	48	16%
P7	Has there been any change in behaviour towards family members?	1. Avoid hugging	1	99	33%
		2. Avoid kissing	1	108	36%
		3. Avoid sharing food from the same plate	1	96	32%
		4. Avoid sleeping together	1	87	29%
		5. No change	0	111	37%
P8	Do you wear a mask when you go outside your home to prevent contracting and spreading of COVID-19?	1. Always	2	138	46%
		2. Most of the times	1	129	43%
		3. Do not believe in it	0	33	11%
P9	Any change in eating habits to prevent contracting COVID-19?	1. Eat more of lemon	1	78	26%
		2. Increased garlic in the food	1	57	19%
		3. Drink hot water	1	75	25%
		4. Avoid eating from outside	1	39	13%
		5. No change	0	51	17%
P10	Any precaution that you take while coughing to prevent spreading of COVID-19?	1. Use tissue	1	39	19%
		2. Use Handkerchief	1	99	33%
		3. Bent of elbow	1	172	44%
		4. Use hand	0	12	4%
P11	Given all the options, how would you prefer to feed your baby after birth?	1. Breastfeed	2	195	65%
		2. Expressed breast milk	1	51	17%
		3. Formula feed	1	56	18%
P12	Which mask do you wear to prevent contracting and spreading covid?	1. N95	NA	201	67%
		2. Cloth		18	9%
		3. Surgical mask		51	17%
		4. 2 surgical mask or 1 cloth and 1 surgical mask		21	7%
P13	Side effects of covid vaccination done during pregnancy?	1. Fever	NA	129	43%
		2. Pain at injection site		117	39%
		3. Bleeding per vaginum		21	7%
		4. Pain abdomen		18	6%
		5. Vomiting		27	9%
		6. Abortion		9	3%
		7. Diarrhea		15	5%
		8. More than 2 of above		144	48%
P14	Have you been vaccinated during pregnancy?	1. Yes	NA	34	34%
		2. No		66	66%

Fear of side effects from the vaccine is the main reason for vaccine hesitancy amongst most of the non-vaccinated study population (70%). 79% would prefer home quarantine over quarantine centres and hospitals. In our study majority of the respondents (88%) believed that hospital delivery should be planned for COVID-19-positive pregnant females. 78% of respondents feel that normal delivery should be preferred over LSCS (19%) for COVID-19-positive pregnant females. Only half of the study population (53%) feel that breastfeeding would be an option for a baby born to a COVID-19 mother and the rest would prefer alternate feeds like expressed breast milk (22%) and formula feed (24%).

To prevent COVID-19, 89% of people were practicing COVID-19-appropriate behaviour like wearing a mask (85%), social distancing (83%), avoiding crowded areas (83%) regular hand washing (88%). Around 3% of respondents felt that COVID-19 vaccination in pregnancy can lead to abortion while the majority feel that fever and pain at the injection site will be the most common side effects.

The overall mean score for knowledge was 22.54 which is above the median score of 10 depicting that the response population in our study has adequate knowledge regarding COVID-19 disease, steps for prevention, and vaccination during pregnancy (Table 5).

**Table 5:** Comparison of baseline characteristics of study participants and mean knowledge and practice score using ANOVA or t-test Value for p<0.05 is significant, p<0.01 is more significant

	Knowledge			Practice		
	Mean	SD	p-value	Mean	SD	p-value
Overall	22.54	5.22		23.91	6.72	
Education						
Illiterate	20	0.82	0.147	20.25	2.87	0.09
Tenth	20	4.24		20	5.66	
Twelth	18.28	2.33		18.3	2.26	
Graduate	18.49	2.3		19.05	2.91	
PG	19.5	1.4		20.2	2.4	
Occupation						
Student	19.75	0.51	<0.0001*	19.85	1.94	<0.0001*
Home Maker	18.71	3.12		19	3.14	
Daily Wage Worker	19	2.83		20.25	4.35	
Business Person	17.25	2.87		17	1.31	
Salaried	18.43	2.13		19.29	3.1	
Work from home	16.9	2.52		17.32	2.48	

The overall mean score for practice was 23.91 which is above the median score depicting that the response population is practicing methods enough to prevent COVID-19.

The overall correlation of knowledge and practice is given in Table 6 shows a positive correlation between overall knowledge and practice, i.e., the people who know COVID-19, mode of transmission, signs, and symptoms, and methods of prevention are following methods of prevention like a mask, hand washing (r= 0.095, p=0.097) decrease outgoing (r=0.494, p=<0.0001). There is a correlation between knowledge and practice of dietary habits which is an increased intake of lemons ginger and garlic (0.571, p=<0.0001). Vaccination also shows a positive correlation (r=0.199, p=0.001). The positive correlation is more in the educated group of the population and increases further with the level of education and occupation.

**Table 6:** Pearson’s correlation between the study variable and different domains of knowledge and practices Value for p<0.05 is significant, p<0.01 is more significant.

	Correlation	p-value
Overall Knowledge vs Practice	0.939	<0.0001*
K1 VS P2	0.095	0.097
K4 VS P3	0.494	<0.0001*
K2 VS P5	0.659	<0.0001*
K3 VS P6	0.435	<0.0001*
K9 VS- P9	0.571	<0.0001*
K12 VS P11	0.833	<0.0001*
K7 VS P14	0.199	0.001*

## Discussion

This study was conducted to provide insight into the knowledge, attitude, and practices amongst pregnant women and professional support staff involved in the management of pregnant patients towards COVID-19 infection and vaccination. Pregnant women form a uniquely vulnerable group as a result of immunological suppression during pregnancy and active measures should be taken to prevent COVID-19 infection in this subgroup as COVID-19 infection is associated with both maternal and foetal complications. This step is crucial, particularly in developing nations like India where the health infrastructure and support are not as advanced as in developed countries and are not readily accessible to every pregnant female.

In our study participants had an overall correct knowledge rate of 68.3%, suggesting that the majority of pregnant women had correct coronavirus knowledge but the previous study in China amongst the general population showed a higher correct knowledge rate (90%) [8]. Overall, the mean knowledge score (22.54) of the participants in this study was adequate which is in agreement with the knowledge level amongst pregnant women in the previously conducted study [9]. The adequate knowledge in our study population could be attributed to magnificent efforts by the Government of India in spreading information and increasing awareness amongst the population since the beginning of the pandemic using means of mass communication like TV, radio, internet, etc.

In our study, a significant predictor of knowledge and practice was occupation (p-value = <0.0001) and any significant correlation of knowledge score with education was not evident which is in contrast to previous studies conducted globally [8,9]. In our study, the respondents reported fever (65%), cough (60%), and breathlessness (56%) as common symptoms of COVID-19 but pregnant women were comparatively less aware of weakness/myalgia (45%) which is similar to the results by Erfani et al. [10] conducted in Iran and this could be possible as myalgia and weakness are considered as common physiological symptoms in the pregnant population.

In our study, most of the participants (83%) believed that COVID-19 infection poses an additional risk of infection during pregnancy due to its effect on the foetus (5%), delivery-related complications (4%), antenatal complications (6%) or all of above (41%). Amongst enrolled participants, 44% thought that more research was required on this topic while 6% did not answer. These concerns amongst the study population can lead the policy-makers towards unnecessary apprehension present amongst pregnant mothers as revealed by other studies [11,12]. 17% of the respondents were aware that hydroxychloroquine was a treatment modality for COVID-19 whereas 80% responded that the available vaccine was the treatment of choice.

More than half (53%) of the participants believed that breastfeeding was the feeding option of choice for COVID-19-positive mothers followed by expressed breast milk (22%). Also, the majority of the respondents (78%) were in favour of normal vaginal delivery as a mode of delivery in COVID-19-positive pregnant females.

Overall, the respondents showed a positive attitude regarding a visit to the health care facility when suspected to have COVID-19-related symptoms (73%). 86% of pregnant women believed that India's fight against this health emergency is still not over. These findings are in line with recent studies by Zhong et al. and Al-Hanawi et al. where the results showed positive attitudes amongst the general public [8,13]. Positive attitudes and utmost confidence in control of COVID-19 amongst the general population could be a reflection of the government's planned and relentless actions, stringent successful steps such as nationwide lockdown, and suspension of routine activities such as schools and universities along with domestic and international air travel [14].

There is a common global consensus that educated people comply better with preventive and treatment measures. In our study as expected, there was a significant statistical correlation (p-value <0.0001) between the overall knowledge score and the practices followed by our respondents. The participants enrolled in our study frequently practiced increased safety measures such as wearing masks (85%), avoiding crowded places (83%), avoiding handshakes (80%), social distancing (80%), and washing vegetables before storing them (52%) which could be readily explained by their awareness of mode of spread of COVID-19. It is similar to the level of practice observed amongst pregnant women in the study conducted by Kamal et al. [9].

The study results could help various authorities in designing policies directed towards pregnant women with a special focus on those with low KAP (e.g., non-healthcare workers) who are at much higher risk of contracting the disease.

The areas that need to be focused extensively include the duration of handwashing and the preferred method of breastfeeding in COVID-19-affected mother. Pregnancy is a very important aspect that requires extra attention and dissemination of correct and scientifically validated information regarding knowledge and right practices that will help diminish the anxiety and apprehension amongst pregnant women and their relatives and will enhance positive attitudes amongst all healthcare workers involved in the management of pregnant women.

This study will provide insight into KAP amongst pregnant women and healthcare workers involved in the management of COVID-19 in target population. The questionnaire had been designed based on standard WHO resources [15] and it evaluated various aspects of COVID-19 disease. Correct knowledge, right practices, and positive attitude amongst the general population are the only pillars of prevention with novel coronavirus variants which are emerging, rising number of cases, and an uncertain future [15]. Due to rapidly changing evidence on this topic, answers to the questionnaire may change over time and new options may be included. Furthermore, it is a single-centre study conducted at a periurban centre and may not be generalised to the entire population. In the future, multicentric research amongst both urban and rural population is warranted for better KAP assessment of pregnant women and other healthcare workers.

Strength and limitations

The study was conducted in a periurban population with limited access to healthcare. The respondents included pregnant women, and patient care attendants who had less formal education and they rely mostly on means of mass communication for their knowledge and awareness. The study needs to be done amongst patients in all strata of society and should have a larger sample size so that the results can be extrapolated to the general population. Any pandemic should be fought with participation from all sections of society where there is no place for half-baked knowledge and misinformation.

## Conclusions

This study demonstrated that the majority of the pregnant women and healthcare workers involved in the management of pregnant women had satisfactory knowledge and positive attitude and were following appropriate practices regarding COVID-19 but further focused efforts in creating awareness amongst various subpopulations should be continued. As India is battling the fourth coronavirus wave and in the absence of a definitive cure, strengthening health policies directed at ensuring the safety of pregnant women and newborn babies should be prioritized with a special focus on significant gaps in KAP.
